# Supplementary material for: Association between HLA Class II Alleles/Haplotypes and Genomic Ancestry in Brazilian Patients with Type 1 Diabetes: A Nationwide Exploratory Study
Source: Genes (Basel). 2023 Apr 27;14(5):991. doi: 10.3390/genes14050991 (PMC10218425; doi:10.3390/genes14050991)
Supplement: Supplementary file 1 [file genes-14-00991-s001.zip › genes-2341716-supplementary.pdf]

Supplementary Materials

Table S1. Distribution of the HLA-DRB1~DQA1~DQB1 haplotypes (risk and protective) in patients with type 1 diabetes and individuals without type 1 diabetes. (Reference 7).

**Haplotypes (*DRB1*~DQA1*~DQB1**)**	**Individuals with T1D n (%)**	**Individuals without T1D n (%)**	**OR**	**95% CI**	**P**
**Risk haplotypes**					
03:01~05:01~02:01	590 (28.95%)	671 (6.56%)	5.8	5.13-6.57	.00000
04:01~03:01~03:02	102 (4.99%)	81 (0.79%)	6.6	4.91-8.87	.00000
04:02~03:01~03:02	130 (6.39%)	199 (1.94%)	3.43	2.74-4.31	.00000
04:04~03:01~03:02	69 (3.39%)	100 (0.98%)	3.55	2.60-4.84	.00000
04:05~03:01~03:02	205 (10.06%)	210 (2.05%)	5.34	4.37-6.51	.00000
09:01~03:01~02:02	58 (2.86%)	61 (0.60%)	4.88	3.40-7.02	.00000
**Protective haplotypes**					
01:02~01:01~05:01	54 (2.65%)	578 (5.65%)	0.45	0.34-0.60	.00000
03:02~04:01~04:02	10 (0.49%)	306 (2.99%)	0.16	0.08-0.30	.00000
04:11~03:01~03:02	14 (0.69%)	258 (2.52%)	0.27	0.16-0.46	.00000
07:01~02:01~02:02	125 (6.12%)	1112 (10.87%)	0.54	0.44-0.65	.00000
07:01~02:01~03:03	4 (0.20%)	166 (1.63%)	0.12	0.04-0.32	.00000
08:02~04:01~04:02	6 (0.29%)	139 (1.36%)	0.21	0.09-0.49	.00004
08:03~06:01~03:01	1 (0.05%)	68 (0.66%)	0.07	0.01-0.53	.00012
11:01~01:02~06:02	8 (0.39%)	327 (3.19%)	0.12	0.06-0.24	.00000
13:01~01:03~06:03	43 (2.11%)	676 (6.60%)	0.3	0.22-0.42	.00000
14:02~05:01~03:01	1 (0.05%)	227 (2.22%)	0.02	0.003-0.15	.00000
14:54~01:01~05:03	1 (0.05%)	79 (0.77%)	0.06	0.009-0.45	.00001
15:01~01:02~06:02	16 (0.79%)	414 (4.04%)	0.19	0.11-0.31	.00000
15:03~01:02~06:02	18 (0.88%)	503 (4.92%)	0.17	0.11-0.28	.00000
16:01~01:02~05:02	17 (0.83%)	212 (2.07%)	0.4	0.24-0.65	.00014
Others	159 (7.80%)	660 (6.45%)	1.23	1.03-1.47	.02560

T1D=type 1 diabetes mellitus; n=number of individuals; OR=odds ratio; CI=confidence interval; Sixty-eight haplotypes with total number in patients plus controls >18 were included (0.3%). P required for statistical significance after Bonferroni correction for multiple tests < 0.00074. Rare alleles were included in others.

Table S2. ROC curve analysis of global genomic ancestry of HLA-DRB1* alleles.

Variables	AUC	95% CI	P	Threshold of ancestry percentage	Sensitivity (%)	Specificity (%)
03:01						
**EUR**	**0.533**	**0.505 to 0.562**	**0.021**	**> 58.4%**	**63.8**	**42.8**
AFR	0.531	0.503 to 0.559	0.032	< 15.9	49.7	55.7
NAM	0.512	0.484 to 0.540	0.402	< 5.95	30.8	73.1
04:05						
EUR	0.504	0.469 to 0.538	0.832	< 70.1	62.4	41.5
AFR	0.500	0.465 to 0.535	0.998	> 23.7	39.7	63.6
NAM	0.510	0.476 to 0.544	0.566	> 12.3	53.5	51.6
07:01						
EUR	0.522	0.483 to 0.561	0.283	> 70.3	46.0	60.6
AFR	0.504	0.464 to 0.544	0.842	> 3.1	88.9	14.2
NAM	0.540	0.501 to 0.579	0.050	< 11.0	54.0	55.1
04:02						
**EUR**	**0.545**	**0.504 to 0.586**	**0.037**	**> 46.3**	**86.8**	**22.1**
AFR	0.528	0.488 to 0.568	0.189	< 34.1	86.3	22.0
NAM	0.524	0.483 to 0.565	0.261	< 15.2	66.2	42.9
04:01						
**EUR**	**0.558**	**0.514 to 0.603**	**0.009**	**> 91.6**	**23.2**	**88.3**
AFR	0.576	0.532 to 0.620	<0.001	< 3.9	28.4	84.1
NAM	0.506	0.461 to 0.550	0.795	< 6.5	34.5	69.8
04:04						
EUR	0.501	0.453 to 0.550	0.957	< 84.5	85.6	23.1
AFR	0.513	0.466 to 0.561	0.610	< 30.3	81.8	26.9
**NAM**	**0.552**	**0.500 to 0.604**	**0.047**	**> 7.9**	**75.0**	**37.5**
01:01						
EUR	0.533	0.480 to 0.585	0.215	> 52.6	77.7	30.2
AFR	0.552	0.498 to 0.605	0.051	< 4.5	30.0	81.8
NAM	0.514	0.460 to 0.567	0.600	< 13.7	59.2	46.4
13:02						
EUR	0.537	0.481 to 0.592	0.191	< 30.6	14.8	92.9
AFR	0.547	0.490 to 0.604	0.094	> 44.2	20.0	89.9
NAM	0.510	0.455 to 0.564	0.731	< 17.9	69.6	34.9
01:02						
EUR	0.507	0.445 to 0.569	0.804	> 90.4	23.1	85.6
AFR	0.513	0.454 to 0.573	0.649	> 34.0	27.9	79.3
NAM	0.525	0.466 to 0.583	0.402	< 3.4	25.0	81.3
09:01						
EUR	0.677	0.621 to 0.733	<0.001	< 47.7	53.1	79.3
**AFR**	**0.679**	**0.622 to 0.736**	**<0.001**	**> 26.0**	**60.4**	**69.0**
NAM	0.540	0.483 to 0.597	0.185	> 2.8	94.8	16.1
13:01						
EUR	0.503	0.437 to 0.569	0.928	< 43.2	23.2	82.5
AFR	0.507	0.443 to 0.571	0.832	> 52.5	11.0	94.1
NAM	0.514	0.448 to 0.580	0.667	< 12.0	54.9	51
11:01						
EUR	0.527	0.449 to 0.605	0.477	< 36.1	21.3	88.9
AFR	0.544	0.467 to 0.621	0.244	> 37.8	27.9	83.8
NAM	0.503	0.433 to 0.574	0.927	< 12.3	57.4	49.7
08:01						
**EUR**	**0.583**	**0.513 to 0.653**	**0.032**	**> 50.8**	**89.7**	**27**
AFR	0.540	0.467 to 0.613	0.303	< 15.5	58.6	54.1
NAM	0.578	0.508 to 0.647	0.044	< 19.8	86.2	30.8
16:01						
EUR	0.542	0.465 to 0.619	0.365	> 54.2	80.0	32.8
AFR	0.554	0.477 to 0.632	0.241	< 36.6	97.5	18.3
NAM	0.530	0.439 to 0.620	0.517	> 29.4	25.0	86.9
15:01						
EUR	0.533	0.428 to 0.638	0.495	< 50.5	41.7	74.3
AFR	0.516	0.418 to 0.613	0.747	< 17.8	66.7	49.4
NAM	0.547	0.452 to 0.643	0.330	> 21.2	38.9	74.0
10:01						
EUR	0.535	0.428 to 0.641	0.503	< 64.8	65.6	50.5

AFR	0.587	0.478 to 0.695	0.093	> 19.8	65.6	55.3
NAM	0.619	0.519 to 0.718	0.021	< 6.2	53.1	70.7
08:04						
EUR	0.656	0.556 to 0.756	0.005	< 53.7	70.4	69.0
**AFR**	**0.614**	**0.505 to 0.723**	**0.042**	**> 16.5**	**74.1**	**48.6**
NAM	0.584	0.468 to 0.700	0.135	> 17.8	59.3	65.5
04:11						
EUR	0.537	0.427 to 0.647	0.527	< 64.4	60.0	50.8
AFR	0.517	0.426 to 0.608	0.775	> 6.1	92.0	24.0
NAM	0.582	0.468 to 0.696	0.159	> 11.3	72.0	47.8
15:03						
EUR	0.697	0.616 to 0.778	<0.001	< 55.2	69.2	65.8
**AFR**	**0.690**	**0.597 to 0.783**	**<0.001**	**> 13.4**	**88.5**	**41.8**
NAM	0.579	0.467 to 0.692	0.164	> 7.9	84.6	36.8
11:04						
EUR	0.500	0.406 to 0.594	0.999	< 77.1	80.0	31.4
AFR	0.510	0.420 to 0.601	0.858	> 6.0	92.0	23.9
NAM	0.517	0.401 to 0.632	0.774	> 14.1	56.0	55.4
12:01						
EUR	0.511	0.394 to 0.627	0.859	< 63.3	58.3	52.9
AFR	0.580	0.466 to 0.694	0.178	< 7.0	41.7	74.2
NAM	0.593	0.470 to 0.715	0.119	> 20.9	50.0	73.3
16:02						
EUR	0.706	0.598 to 0.815	<0.001	< 56.3	78.3	64.6
**AFR**	**0.678**	**0.576 to 0.780**	**0.003**	**> 15.4**	**87.0**	**46.7**
**NAM**	**0.621**	**0.511 to 0.730**	**0.046**	**> 26.2**	**39.1**	**83.1**
03:02						
EUR	0.716	0.594 to 0.837	<0.001	< 48.7	63.6	76.7
**AFR**	**0.649**	**0.516 to 0.782**	**0.016**	**> 48.3**	**36.4**	**92.4**
NAM	0.597	0.474 to 0.719	0.119	> 11.3	72.7	47.8
13:03						
EUR	0.577	0.434 to 0.721	0.223	< 46.5	47.6	79.1
AFR	0.611	0.457 to 0.765	0.080	> 36.5	52.4	82.1
NAM	0.626	0.510 to 0.742	0.046	< 11.4	81.0	52.6
04:07						
EUR	0.561	0.470 to 0.652	0.360	< 74.3	89.5	34.6
AFR	0.529	0.419 to 0.639	0.662	< 20.5	73.7	43.5
**NAM**	**0.652**	**0.535 to 0.769**	**0.022**	**> 6.5**	**94.7**	**31.0**
11:02						
EUR	0.589	0.448 to 0.730	0.192	< 58.1	61.1	61.1
**AFR**	**0.636**	**0.488 to 0.783**	**0.048**	**> 35.9**	**50.0**	**81.5**
NAM	0.525	0.442 to 0.609	0.714	< 19.9	94.4	30.3
04:03						
EUR	0.501	0.376 to 0.625	0.993	< 60.2	52.9	57.7
AFR	0.505	0.374 to 0.637	0.940	> 14.0	70.6	42.9
NAM	0.509	0.382 to 0.635	0.903	> 2.9	94.1	15.8
08:02						
EUR	0.650	0.510 to 0.791	0.045	< 55.6	73.3	65.5
AFR	0.546	0.406 to 0.686	0.543	> 14.8	73.3	45.1
**NAM**	**0.648**	**0.495 to 0.801**	**0.048**	**> 23.4**	**60.0**	**78.5**
14:02						
EUR	0.539	0.406 to 0.671	0.673	< 75.6	90.0	33.2
AFR	0.586	0.429 to 0.743	0.348	> 25.2	60.0	66.2
NAM	0.503	0.369 to 0.638	0.971	> 3.6	100	19.9
04:08						
EUR	0.536	0.352 to 0.719	0.698	> 68.3	60.0	56.0
AFR	0.533	0.367 to 0.699	0.720	> 8.9	90.0	31.0
NAM	0.582	0.429 to 0.735	0.370	< 9.6	70.0	58.0

ROC=Receiver operating characteristic. AUC=Area under the curve. 95% CI=95% confidence interval. EUR=European ancestry percentage. AFR=African ancestry percentage. NAM=Native American ancestry percentage.

Table S3. ROC curve analysis of global genomic ancestry of HLA-DQA1 alleles.

Variables HLA-DQA1*	AUC	95% CI	P	Threshold of ancestry percentage	Sensitivity (%)	Specificity (%)
03:01						
EUR	0.514	0.485 to 0.543	0.357	< 54.6	35.2	69.1
AFR	0.507	0.478 to 0.536	0.638	> 15.9	54.5	49.3
NAM	0.523	0.494 to 0.552	0.121	> 9.0	61.8	44.2
05:01						
**EUR**	**0.533**	**0.505 to 0.562**	**0.022**	**> 60.9**	**58.8**	**47.5**
AFR	0.528	0.500 to 0.557	0.053	< 24.2	66.5	39.1
NAM	0.514	0.486 to 0.543	0.326	< 8.5	40.4	63.5
01:01						
EUR	0.518	0.480 to 0.557	0.332	> 88.3	23.2	83.4
AFR	0.513	0.475 to 0.551	0.479	< 4.8	26.1	81.1
NAM	0.529	0.491 to 0.567	0.127	< 13.4	58.1	48.2
01:02						
EUR	0.566	0.526 to 0.605	0.001	< 80.2	81.6	29.1
**AFR**	**0.551**	**0.511 to 0.591**	**0.012**	**> 37.8**	**25.1**	**84.9**
NAM	0.533	0.493 to 0.572	0.104	> 5.4	79.5	28.5
02:01						
EUR	0.528	0.488 to 0.569	0.177	> 64.6	56.6	50.7
AFR	0.505	0.464 to 0.546	0.814	< 18.1	56.6	49.1
NAM	0.542	0.502 to 0.583	0.043	< 11.0	54.3	55.1
04:01						
EUR	0.551	0.491 to 0.610	0.076	< 58.1	53.6	61.9
AFR	0.530	0.473 to 0.587	0.290	> 12.3	69.1	39.0
NAM	0.528	0.469 to 0.587	0.331	> 23.3	30.0	78.6
01:03						
EUR	0.563	0.496 to 0.631	0.063	> 92.9	22.7	90.0
AFR	0.545	0.480 to 0.611	0.187	< 4.3	28.0	82.0
NAM	0.557	0.488 to 0.625	0.098	< 4.5	34.7	76.5

ROC=Receiver operating characteristic. AUC=Area under the curve. 95% CI=95% confidence interval. EUR=European ancestry percentage. AFR=African ancestry percentage. NAM=Native American ancestry percentage.

Table S4. ROC curve analysis of global genomic ancestry of HLA-DQB1* alleles.

Variables HLA-DQB1*	AUC	95% CI	P	Threshold of ancestry percentage	Sensitivity (%)	Specificity (%)
02:01						
**EUR**	**0.536**	**0.508 to 0.564**	**0.013**	**> 58.2**	**64.3**	**42.6**
AFR	0.532	0.503 to 0.560	0.029	< 15.9	49.6	55.5
NAM	0.514	0.485 to 0.542	0.342	< 5.9	30.7	73.0
03:02						
**EUR**	**0.529**	**0.501 to 0.557**	**0.045**	**> 44.9**	**83.6**	**22.3**
AFR	0.542	0.514 to 0.570	0.004	< 32.7	81.8	27.7
NAM	0.524	0.496 to 0.552	0.099	> 9.0	62.7	43.9
02:02						
EUR	0.510	0.475 to 0.545	0.587	< 37.5	16.6	88.6
**AFR**	**0.543**	**0.508 to 0.579**	**0.015**	**> 30.7**	**32.8**	**75.9**
NAM	0.546	0.512 to 0.580	0.010	< 10.7	53.3	56.7
05:01						
EUR	0.512	0.474 to 0.550	0.523	> 90.4	20.1	86.1
AFR	0.501	0.462 to 0.539	0.978	< 4.8	25.3	81.0
NAM	0.539	0.501 to 0.576	0.039	< 10.3	50.7	57.3
03:01						
EUR	0.540	0.496 to 0.584	0.068	< 56.3	45.5	65.3
**AFR**	**0.547**	**0.503 to 0.592**	**0.031**	**> 35.3**	**29.3**	**81.9**
NAM	0.507	0.464 to 0.549	0.763	< 12.3	54.5	50.0
04:02						
EUR	0.550	0.488 to 0.612	0.088	< 58.1	53.4	61.8
AFR	0.522	0.464 to 0.580	0.449	> 6.3	81.6	24.7
NAM	0.536	0.475 to 0.597	0.222	> 23.3	31.1	78.7
06:04						
EUR	0.521	0.457 to 0.585	0.520	< 72.6	70.7	37.2
AFR	0.538	0.473 to 0.603	0.246	> 41.8	22.0	87.2
NAM	0.509	0.445 to 0.572	0.790	< 16.7	69.5	38.0
06:03						
EUR	0.556	0.485 to 0.627	0.112	> 93.1	21.4	90.3
AFR	0.550	0.483 to 0.617	0.154	< 27.7	80.0	30.5
NAM	0.539	0.466 to 0.612	0.270	< 4.5	34.3	76.5
06:02						
EUR	0.608	0.535 to 0.681	0.005	< 51.7	50.0	72.7
**AFR**	**0.594**	**0.519 to 0.669**	**0.015**	**> 37.8**	**37.9**	**84.2**
NAM	0.535	0.459 to 0.611	0.362	> 6.7	77.6	32.3
05:02						
EUR	0.502	0.432 to 0.571	0.967	> 41.8	91.2	16.7
AFR	0.517	0.447 to 0.588	0.654	< 20.4	63.2	43.9
NAM	0.549	0.476 to 0.621	0.212	> 4.7	86.0	25.2
03:03						
EUR	0.583	0.496 to 0.671	0.066	< 50.6	42.9	74.3
AFR	0.557	0.467 to 0.647	0.206	> 25.2	47.6	66.4
NAM	0.549	0.462 to 0.636	0.277	> 13.9	54.8	54.8
06:09						
EUR	0.595	0.472 to 0.718	0.143	< 64.1	70.0	51.3
AFR	0.531	0.394 to 0.668	0.636	> 32.8	35.0	77.5
NAM	0.606	0.486 to 0.726	0.103	> 16.6	60.0	62.3
05:03						
EUR	0.507	0.380 to 0.633	0.930	< 68.8	73.3	43.1
AFR	0.509	0.376 to 0.642	0.906	< 39.5	100	15.3
NAM	0.536	0.386 to 0.685	0.634	> 34.5	20.0	92.6
05:07						
EUR	0.631	0.475 to 0.787	0.134	< 58.6	72.7	60.4
AFR	0.521	0.362 to 0.680	0.810	> 9.85	81.8	32.8
**NAM**	**0.702**	**0.553 to 0.852**	**0.021**	**> 23.0**	**63.6**	**77.4**

ROC=Receiver operating characteristic. AUC=Area under the curve. 95% CI=95% confidence interval. EUR=European ancestry percentage. AFR=African ancestry percentage. NAM=Native American ancestry percentage.

Table S5. Descriptive statistics of global genomic ancestry percentage according to HLA-DRB1 alleles.

HLA-	European ancestry (%)				African ancestry (%)				Native American ancestry (%)			
DRB1*	Mean	±SD	Minimum	Maximum	mean	±SD	Minimum	Maximum	Mean	±SD	Minimum	Maximum
03:01	65.8	20.8	5.5	99.2	19.7	16.1	0.4	88.7	14.5	11.7	0.4	68.5
04:05	64.2	21.1	4.6	99.1	20.8	17.2	0.2	88.7	15.0	11.6	0.5	69.5
07:01	65.3	21.1	2.3	98.8	20.9	16.8	0.3	77.7	13.8	11.8	0.5	60.7
04:02	67.7	20.1	5.7	98.8	18.7	15.3	0.6	83.1	13.6	10.7	0.5	53.3
04:01	68.2	21.8	12.9	98.7	17.3	15.6	0.2	70.3	14.5	11.8	0.6	60.7
04:04	64.4	20.4	7.2	98.6	18.8	13.8	0.7	60.1	16.8	12.3	0.7	50.5
01:01	66.3	22.4	7.9	99.2	18.7	18.4	0.4	87.7	15.0	13.0	0.4	68.5
13:02	60.7	23.3	11.5	98.1	24.6	19.8	0.6	79.7	14.7	12.6	0.6	69.5
01:02	64.3	23.7	2.3	98.5	21.8	17.6	0.3	72.2	13.8	11.2	0.8	52.2
09:01	50.4	22.2	8.2	96.8	32.4	19.8	1.0	85.7	17.2	13.8	1.1	62.6
13:01	63.4	22.5	4.6	97.0	22.3	18.9	1.2	78.7	14.3	11.9	0.7	50.0
11:01	60.9	24.3	5.7	97.1	24.5	19.2	0.6	73.1	14.6	11.5	0.6	51.2
08:01	70.4	18.7	28.0	97.9	18.1	14.3	1.0	61.4	11.5	9.2	0.6	35.6
16:01	67.7	17.0	31.3	98.5	16.4	11.6	0.3	41.2	15.8	11.9	1.2	36.0
15:01	61.6	24.3	17.4	97.8	21.0	19.5	0.9	75.6	17.4	14.6	1.3	68.6
10:01	61.2	24.0	7.9	99.1	28.4	23.0	0.4	87.7	10.4	9.9	0.5	38.6
08:04	53.7	20.1	10.0	97.6	27.8	18.8	1.0	70.5	18.5	13.1	0.5	53.3
04:11	60.3	21.9	15.1	96.1	20.9	14.1	1.5	66.2	18.8	12.9	1.1	51.2
15:03	50.7	16.0	20.8	79.2	31.7	17.0	2.1	63.5	17.6	11.2	0.5	35.6
11:04	64.7	17.2	26.0	96.9	20.0	14.2	1.6	68.3	15.3	11.9	0.5	41.5
12:01	63.8	21.2	27.7	97.1	16.7	16.0	1.1	58.3	19.5	14.9	0.9	62.6
16:02	48.4	20.5	22.0	95.5	32.3	18.0	1.2	66.1	19.3	12.0	3.3	40.8
03:02	45.8	24.3	17.0	94.2	36.0	26.3	1.6	75.9	18.2	13.4	1.4	41.0

13:03	58.2	25.2	25.9	96.3	31.2	24.2	1.8	65.7	10.6	11.7	1.1	44.4
04:07	61.0	13.3	39.1	84.8	17.7	12.0	1.6	39.9	21.3	13.5	1.6	46.3
11:02	56.6	24.7	15.2	96.1	31.4	22.2	0.9	67.9	12.0	6.1	3.0	26.0
04:03	65.3	18.1	40.9	95.4	20.2	14.4	0.7	48.2	14.5	10.9	2.0	39.9
08:02	55.1	20.8	26.9	97.1	22.4	16.4	1.2	61.2	22.5	17.6	1.7	68.6
14:02	61.8	14.4	41.8	92.3	24.8	14.5	3.9	50.8	13.3	7.5	3.7	25.6
04:08	66.4	22.8	27.7	96.0	22.8	18.7	2.6	63.1	10.8	8.1	1.4	22.7

Table S6. Descriptive statistics of global genomic ancestry percentage according to HLA-DQA1 alleles.

HLA-DQA1*	European ancestry (%)				African ancestry(%)				Native American ancestry(%)			
	**Mean**	**±SD**	**Minimum**	**Maximum**	**mean**	**±SD**	**Minimum**	**Maximum**	**mean**	**±SD**	**Minimum**	**Maximum**
03:01	63.6	21.4	4.6	99.1	21.1	16.9	0.2	88.7	15.2	11.9	0.5	69.5
05:01	65.5	21.0	5.5	99.2	20.1	16.6	0.4	88.7	14.5	11.5	0.4	68.5
01:01	64.9	22.7	2.3	99.2	20.8	18.1	0.3	87.7	14.3	12.1	0.4	68.5
01:02	59.0	22.0	4.6	98.5	24.5	19.2	0.3	79.7	16.5	12.8	0.5	69.5
02:01	65.7	21.2	2.3	98.8	20.5	17.0	0.3	77.7	13.8	11.9	0.5	60.7
04:01	60.5	23.1	10.0	97.9	23.2	18.7	1.0	75.9	16.3	13.5	0.6	68.6
01:03	68.5	20.9	19.6	97.1	18.5	15.8	1.2	69.6	13.0	11.7	0.7	50.0

Table S7. Descriptive statistics of global genomic ancestry percentage according to HLA-DQB1 alleles.

HLA-DQB1*	European ancestry (%)				African ancestry (%)				Native American ancestry (%)			
	**mean**	**±SD**	**Minimum**	**Maximum**	**mean**	**±SD**	**Minimum**	**Maximum**	**mean**	**±SD**	**Minimum**	**Maximum**
02:01	65.9	20.8	5.5	99.2	19.7	16.1	0.4	88.7	14.5	11.6	0.4	68.5
03:02	65.2	20.8	4.6	99.1	19.4	15.7	0.2	88.7	15.4	11.8	0.5	69.5
02:02	62.8	22.3	2.3	98.8	23.6	18.6	0.37	85.7	13.6	11.7	0.6	62.6
05:01	64.2	23.1	2.3	99.2	21.8	19.0	0.3	87.7	14.0	12.0	0.4	68.5
03:01	61.7	22.1	5.7	98.1	24.1	19.2	0.6	71.4	14.2	11.2	0.5	51.2
04:02	60.4	23.5	17.0	97.9	23.0	19.0	1.0	75.9	16.6	13.7	0.6	68.6
06:04	61.5	23.2	11.5	98.1	23.8	18.8	0.6	79.7	14.7	12.3	0.6	63.6
06:03	68.1	21.6	19.6	97.1	18.1	15.9	1.2	69.6	13.8	12.3	0.7	50.0
06:02	56.7	20.5	9.4	97.1	26.7	18.7	1.1	73.1	16.6	13.5	0.5	68.6
05:02	64.7	18.9	22.0	98.5	19.2	14.9	0.3	66.1	16.1	11.0	1.2	37.9
03:03	57.8	21.6	18.6	96.8	24.4	17.7	0.7	63.5	17.8	14.1	1.1	61.0
06:09	58.1	23.1	15.7	94.5	23.2	19.3	0.7	63.3	18.7	13.5	2.3	52.3
05:03	65.8	18.1	31.3	97.2	17.9	12.9	1.3	39.3	16.3	13.7	1.5	47.6
05:07	54.9	18.7	28.5	96.2	21.4	16.6	1.6	59.6	23.7	11.6	2.2	38.4

Table S8. Descriptive statistics of global genomic ancestry percentage according to HLA haplotypes.

haplotype	European ancestry (%)				African ancestry (%)				Native American ancestry (%)			
	mean	±SD	Minimum	Maximum	mean	±SD	Minimum	Maximum	mean	±SD	Minimum	Maximum
Risk haplotypes												
03:01~05:01~02:01	65.6	21.1	5.5	99.2	19.9	16.3	0.4	88.7	14.5	11.7	0.4	68.5
04:01~03:01~03:02	67.1	21.3	12.9	98.7	17.3	14.7	0.2	67.0	15.6	12.0	0.6	60.2
04:02~03:01~03:02	67.7	20.0	15.7	98.8	19.0	15.4	0.6	83.1	13.3	10.3	0.5	53.3
04:04~03:01~03:02	64.7	20.2	7.2	98.6	18.8	13.7	0.7	60.1	16.4	11.9	0.7	50.5
04:05~03:01~03:02	63.5	21.5	4.6	99.1	21.3	17.4	0.2	88.7	15.2	11.7	0.5	69.5
09:01~03:01~02:02	49.8	21.6	8.2	91.4	34.4	19.9	1.0	85.7	15.5	12.4	1.1	62.6
Protective haplotypes												
01:02~01:01~05:01	64.4	24.4	2.3	98.5	22.1	17.8	0.3	72.2	13.5	11.5	0.8	52.2
03:02~04:01~04:02	40.4	22.1	17.0	90.9	38.2	25.8	7.3	75.9	21.4	14.3	1.4	41.0
04:11~03:01~03:02	60.3	21.1	15.1	96.1	20.9	14.2	1.5	66.2	18.7	12.5	1.1	51.2
07:01~02:01~02:02	66.3	21.0	2.3	98.8	20.4	16.8	0.3	77.7	13.3	11.7	0.6	60.7
07:01~02:01~03:03	62.0	20.3	29.1	91.6	19.9	17.0	0.7	57.6	18.1	10.6	4.6	38.9
08:02~04:01~04:02	55.3	22.2	26.9	97.1	23.7	17.5	1.2	61.2	20.9	18.0	1.7	68.6
11:01~01:02~06:02	53.9	23.7	9.4	97.1	36.0	21.5	1.1	73.1	10.2	7.9	0.6	24.8
13:01~01:03~06:03	67.4	21.6	19.6	97.0	18.6	16.1	1.2	69.6	14.0	12.3	0.7	50.0
14:02~05:01~03:01	63.6	15.4	41.8	92.3	22.8	16.7	3.9	50.8	13.7	7.8	3.8	25.6
15:01~01:02~06:02	59.9	23.5	26.3	94.9	21.5	18.5	1.3	65.5	18.6	15.7	1.5	68.6
15:03~01:02~06:02	52.1	15.2	29.3	79.2	29.9	16.3	2.1	54.1	18.0	10.8	0.5	35.6
16:01~01:02~05:02	69.3	16.0	42.3	98.5	15.5	11.6	0.3	41.2	15.2	11.4	1.2	36.0
